# Progress in Psychiatry

**Published:** 1901-12-07

**Authors:** 


### Progress in Psychiatry.

(*Continued from page* 153.)

**Acute Delirium of Meningitis.**—In addition to acute delirium proper, as just described, there are minor forms of cerebral disorder which may occur in comparatively healthy subjects and which partake of a nature of acute delirium. These include the delirium of cerebro-spinal meningitis and tubercular meningitis. Though not requiring asylum care and treatment these diseases come pathologically in the same group of acute cerebral disorders of infective nature, febrile in character, and marked by all the symptoms of acute mental irritation and subsequent exhaustion. The meninges in fatal cases show indications of an acute inflammation with a purulent exudate. The so-called "posterior basic meningitis" of children, which is attended by symptoms of delirium and stupor, is believed by modern observers to be a mild and sporadic form of epidemic cerebro-spinal meningitis (Still[21]). Neither in this affection nor in the epidemic form of cerebro-spinal meningitis is the degree of delirium and excitement equivalent to that of acute delirium proper before mentioned. Psychoses are not confined to any single acute specific fever. They may accompany various fevers, such as typhoid, influenza, pneumonia, scarlet fever, measles, erysipelas, malaria, and the various forms of meningitis, and other rarer affections of an infectious and febrile type. The mental disturbance may precede the pyrexial stage (initial delirium), or it may be co-extensive with the pyrexia (febrile delirium proper), or it may appear after the subsidence of the fever (post-febrile exhaustion psychosis). The first form is rare, but has been typically observed and described in malarial fevers, typhoid, and pneumonia. The second and third forms are observed in influenza and many other febrile affections. If the patient is the subject of cerebral instability or neuropathy, slight fever (100° to 101° Cent.) may be sufficient to provoke delirium or acute hallucinatory mental confusion. In addition to such a constitutional predisposition other causes, such as cerebral traumatism, insolation, or some antecedent physical disease may have left behind a degree of cerebral debility and instability sufficient to give rise to delirium or hallucinatory mental confusion in the patient. The post-febrile form of psychoses (exhaustion-psychoses) stand ætiologically on a special ground of their own. After the conflict between the organism and the pathogenic agencies (microbes) of the fever has ended in victory for the patient and the road to convalesence is entered there may succeed a period of cerebral debility of longer or shorter duration. Any stresses or disturbances at this stage may readily result in mental exhaustion and confusion (asthenic mental confusion) especially in cases with a feeble heart or circulation. On the double basis, therefore, of cerebral and circulatory debility an exhaustion-psychosis may be readily developed by various exciting causes some of which may otherwise seem to be of a trifling nature. Stresses such as want, anxiety, semi-starvation and the like are prone to act very powerfully with such debilitated subjects, and the same may be said of even slight intemperance (whether sexual or alcoholic) at this stage. The resulting psychosis will either be of the asthenic delirious (hallucinatory) type, or more rarely of a stuporous anergic type (acute dementia). The most exhaustive study of post-febrile insanity is that of Kraepelin.[21] He divides it into three forms, viz: (*a*) collapse-delirium, (*b*) acute hallucinatory confusion, and (*c*) acute dementia. Mixed types may also occur in which symptoms of the one kind blend or alternate with the other. (*a*) The exhaustion-delirium is attended by venous congestion and feeble cerebral circulation, lymphatic and leucocytic exudates in the brain and its membranes which have not been fully absorbed, and the occurrence of minute thromboses in the brain from feebleness of circulation and the altered quality of the blood and a degeneration of the capillary and arterial walls. Abnormal oscillations of temperature and great prostration may now succeed, and fleeting hallucinations, insomnia, and restlessness become prominent symptoms. After a period of relative lucidity the patient's mind now becomes confused, he fails to recognise friends and surroundings, illusions of sight first appear and then hallucinations of a horrible and frightful character—both visual and auditory. At this stage excitement is manifested, words and language uttered by the patient show complete confusion and incoherence, restlessness and a desire to get out of bed and to fumble about become marked. Food is refused, and emotions of an erotic or religious character may be displayed. During this loss of flesh takes place daily and rapidly, and grave physical weakness predominates. The temperature sinks to subnormal, the pulse is feeble, muscular irritability and twitchings appear, and death seems imminent. The patient may now sink into a deep sleep lasting a few hours or days, and broken only by occasional delirious mutterings and fumblings, and eventually either begins to regain strength sleep and appetite, or get worse and die of exhaustion. The prognosis is favourable as regards life in three-fourths of all cases of post febrile exhaustion-psychoses, but such recovery of life is generally attended with some degree of persistent mental enfeeblement. When renal complications (albuminuria) or fatty heart and fibroid arteries exist the prognosis is more grave. (*b*) The acute hallucinatory form, though resembling collapse-delirium and having a close affinity with it, has a more acute onset, the prodromal stage not lasting more than a few hours, after which the symptoms of hallucinations and excitement rapidly reach a climax. Painful and terrifying hallucinations are present, as in delirium tremens, and the patient may weep and laugh in total mental confusion, and utter words and phrases of an incoherent character. Tremor is pronounced as in delirium tremens, and intervals of lucidity may momentarily occur. The condition may last from one to two weeks, after which a return to lucidity gets more and more obvious, or a lapse into dementia becomes noticeable. The prognosis is the same as in the previous form of illness. (c) The stuporous or demented type is the one of worst prognosis among the post-febrile psychoses. After a short interval of apparent recovery it is found that the patient is dull and apathetic, lost in a dream-like condition, with cold hands and feeble circulation, failing to recognise friends, expressing no wants, and not speaking at all or uttering a few occasional words and sounds of no import except that they reveal a state of mental vacuity. The patient's attitude is passive and expressive of fatuity, the saliva drips from his lips and chin, he has to be fed, led, clothed and nursed like a helpless infant, his conduct is that of an automaton, without initiative or spontaneity, and occasionally transitory excitement or phrenzy appears and as rapidly disappears. The duration is usually several months, at the end of which slow improvement sets in, or less frequently the patient gradually lapses into permanent and incurable dementia.

[21] Ency·lopædia Media, 1901, article "Meningitis." [22] Archiv. für Psychiatrie und Nervenkrank. 1882.

